# The influence of large cations on the electrochemical properties of tunnel-structured metal oxides

**DOI:** 10.1038/ncomms13374

**Published:** 2016-11-21

**Authors:** Yifei Yuan, Chun Zhan, Kun He, Hungru Chen, Wentao Yao, Soroosh Sharifi-Asl, Boao Song, Zhenzhen Yang, Anmin Nie, Xiangyi Luo, Hao Wang, Stephen M. Wood, Khalil Amine, M. Saiful Islam, Jun Lu, Reza Shahbazian-Yassar

**Affiliations:** 1 Department of Materials Science and Engineering, Michigan Technological University, 1400 Townsend Drive, Houghton, Michigan 49931, USA; 2 Chemical Science and Engineering Division, Argonne National Laboratory, 9700 S. Cass Avenue, Argonne, Illinois 60439, USA; 3 Department of Mechanical and Industrial Engineering, University of Illinois at Chicago, Chicago, Illinois 60607, USA; 4 Department of Chemistry, University of Bath, Bath BA2 7AY, UK; 5 Department of Mechanical Engineering, Michigan Technological University, 1400 Townsend Drive, Houghton, Michigan 49931, USA; 6 Materials Genome Institute and Shanghai Materials Genome Institute, Shanghai University, 99 Shangda Road, Shanghai 200444, China

## Abstract

Metal oxides with a tunnelled structure are attractive as charge storage materials for rechargeable batteries and supercapacitors, since the tunnels enable fast reversible insertion/extraction of charge carriers (for example, lithium ions). Common synthesis methods can introduce large cations such as potassium, barium and ammonium ions into the tunnels, but how these cations affect charge storage performance is not fully understood. Here, we report the role of tunnel cations in governing the electrochemical properties of electrode materials by focusing on potassium ions in α-MnO_2_. We show that the presence of cations inside 2 × 2 tunnels of manganese dioxide increases the electronic conductivity, and improves lithium ion diffusivity. In addition, transmission electron microscopy analysis indicates that the tunnels remain intact whether cations are present in the tunnels or not. Our systematic study shows that cation addition to α-MnO_2_ has a strong beneficial effect on the electrochemical performance of this material.

## Abstract (web summary)

Metal oxides with a tunnelled structure are attractive as charge storage materials for rechargeable batteries and supercapacitors. Here, the authors investigate the role of tunnel cations in governing and enhancing the electrochemical properties of electrode materials.

## Graphical abstract

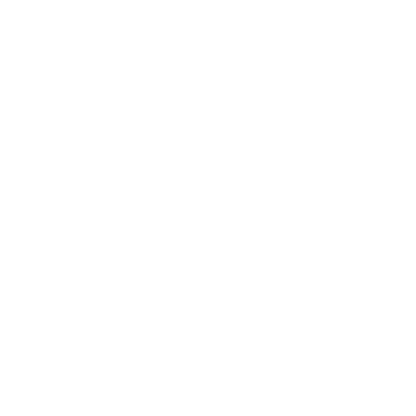

Metal oxides with an internal tunnel structure such as α-MnO_2_, TiO_2_ and β-FeOOH are widely used for charge storage in rechargeable batteries and supercapacitors where charge carriers such as Li^+^, Na^+^ and Mg^2+^ can be reversibly inserted and extracted^1^,^2^,^3^,^4^. Of these oxides, α-MnO_2_, which possesses the typical octahedral molecular sieve structure, is characterized by well-ordered one-dimensional 1 × 1 and 2 × 2 tunnels^5^, as illustrated in Supplementary Fig. 1. The 2 × 2 tunnel (4.6 × 4.6 Å) is large enough to accommodate charge carriers such as Li^+^, Na^+^ and Mg^2+^, enabling fast ion diffusion inside the tunnel cavity^6^. In addition, Mn-based oxides are low cost, environmentally friendly and safer with respect to over-charge conditions compared with Co-based oxide electrodes^7^. Consequently, α-MnO_2_ has been extensively studied as a promising cathode material for lithium (sodium and magnesium) ion batteries^8^,^9^,^10^,^11^, lithium air batteries^12^,^13^ and supercapacitors^14^,^15^,^16^.

Recent research shows that large cations such as K^+^, Ba^2+^ and NH_4_^+^ can be introduced during synthesis of α-MnO_2_ (refs 17, 18). These cations, which partially occupy the tunnel cavities at certain stabilized lattice sites, are expected to interact with the charge carrier (Li^+^, Na^+^ and Mg^2+^) and thus affect the charge storage performance of α-MnO_2_ where tunnel-driven (de)intercalation contributes to the overall capacity^16^,^18^,^19^,^20^. Despite considerable research devoted to improving the charge storage performance of α-MnO_2_, the underlying mechanism by which tunnel cations affect the insertion/extraction of charge carriers is poorly understood.

In the case of Li^+^ being the charge carrier, many researchers believe that the presence of large cations (K^+^, Ba^2+^) inside the 2 × 2 tunnels impedes the diffusion of Li^+^ by physical blocking and repulsive electrostatic forces^18^,^21^,^22^,^23^. Others have pointed out that fast (de)intercalation of Li^+^ requires a stable tunnel structure, and the existence of cations helps to prevent the collapse of the tunnels^24^,^25^. As yet, no conclusive evidence has been provided to clarify whether the tunnel cations promote or impede rapid migration of Li ions. An important factor that contributes to this ambiguity is the difficulty of visualizing these cations and determining their content and position, given the spatial resolution limits of most non-aberration-corrected electron microscopes. It is apparent that there is a pressing need to understand the effect of tunnel cations on charge storage behaviour of materials such as α-MnO_2_ to allow the synthesis methods and properties of such tunnel-structured oxides to be optimized.

In this report, nanowires of K^+^-doped α-MnO_2_ are hydrothermally synthesized and the K^+^ concentration is controlled and differentiated by acid solution treatment. The effect of varying K^+^ concentration on the rate performance of a Li/α-MnO_2_ battery is studied systematically by measuring the electronic conductivity and Li^+^ diffusivity in the α-MnO_2_ cathode. The underlying mechanism is revealed using a powerful combination of aberration-corrected scanning transmission electron microscopy (ACSTEM), transmission electron microscopy (TEM), electrical response measurements of single α-MnO_2_ nanowires, electrochemical impedance spectroscopy (EIS) and density functional theory (DFT) simulations.

## Results

### Structural characterization of α-K_*x*_MnO_2_

**Figure 1:**
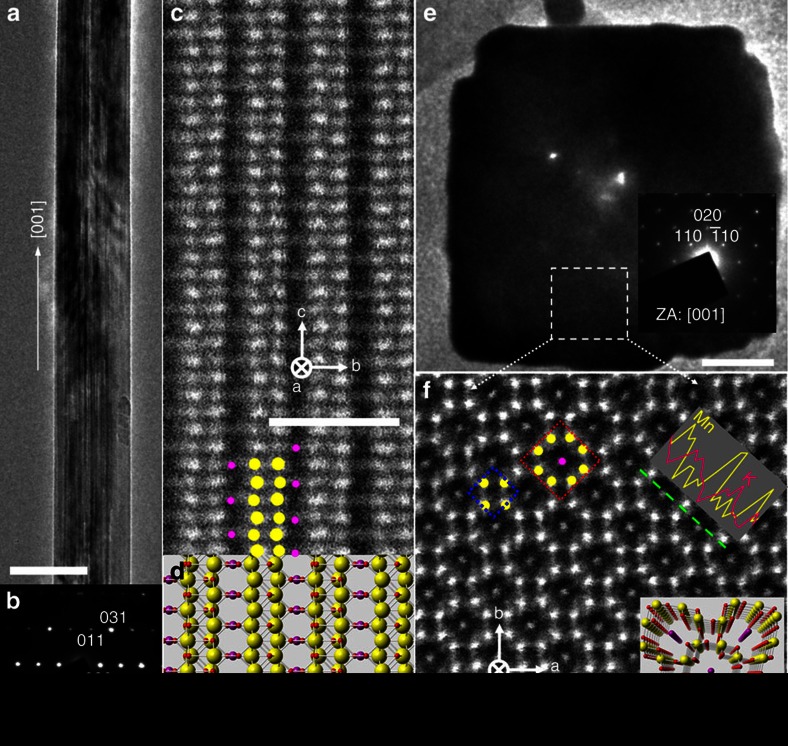
Basic (S)TEM characterization of as-prepared α-MnO_2_ nanowires. (**a**,**b**) A low-magnification TEM image of the as-synthesized α-MnO_2_ nanowire and the corresponding [100] electron diffraction pattern; (**c**,**d**) The [100] HAADF image of the same nanowire with the representative atomic model. The yellow spheres indicate Mn atomic columns, while red and pink represent O and K^+^ ions, respectively. (**e**) A low-magnification [001] TEM image showing one cross-section of α-MnO_2_ nanowire; (**f**) A [001] HAADF image revealing the atomic structure of tunnelled α-MnO_2_ with the red dotted square demarcating a 2 × 2 tunnel and the blue dotted square a 1 × 1 tunnel. The green line indicates the region along which EDS linear scanning was carried out. The bottom inset shows a model of the tunnel structure viewed down the tunnel axis. Scale bars: 50 nm in **a**, 1 nm in **c**,**f**, and 10 nm in **e**.

Hydrothermally synthesized α-MnO_2_ nanowires were characterized using ACSTEM, and the results are shown in Fig. 1. It can be seen from Fig. 1a,b that the α-MnO_2_ nanowire is monocrystalline, growing in the [001] direction with a uniform diameter. The [100] atomic resolution high-angle annular dark field (HAADF) image in Fig. 1c shows the 2 × 2 tunnels (dark stripes) surrounded by two Mn atomic columns (yellow spheres) corresponding to the tunnel walls on each side. It can also be seen that the 2 × 2 tunnels are decorated with ordered K atomic columns (pink spheres) around their centre positions, based on which the [100] atomic model is provided in Fig. 1d. K^+^ ions are successfully introduced during synthesis because excess K^+^ are present in solution to support the 2 × 2 tunnels during their initial formation. The down-tunnel image of one α-MnO_2_ nanowire (Fig. 1e) shows that the nanowire has a square shaped cross-section with four {100} lateral surfaces. The corresponding crystal structure is shown in Fig. 1f, in which 1 × 1 and 2 × 2 tunnels are clearly observed. 1 × 1 tunnels are essentially empty while each 2 × 2 tunnel is found to contain one atomic column, in good agreement with the lateral HAADF image in Fig. 1c. The energy-dispersive spectroscopy (EDS) along the [110] direction shown in Fig. 1f confirmed the tunnel walls to be Mn and the central atoms inside the 2 × 2 tunnels to be K^+^ (Wyckoff 2a positions), from which the atomic model shown in the bottom inset of Fig. 1f was constructed. From these observations, the K^+^ concentration in as-synthesized α-MnO_2_ was determined to be K_0.25_MnO_2_.

**Figure 2:**
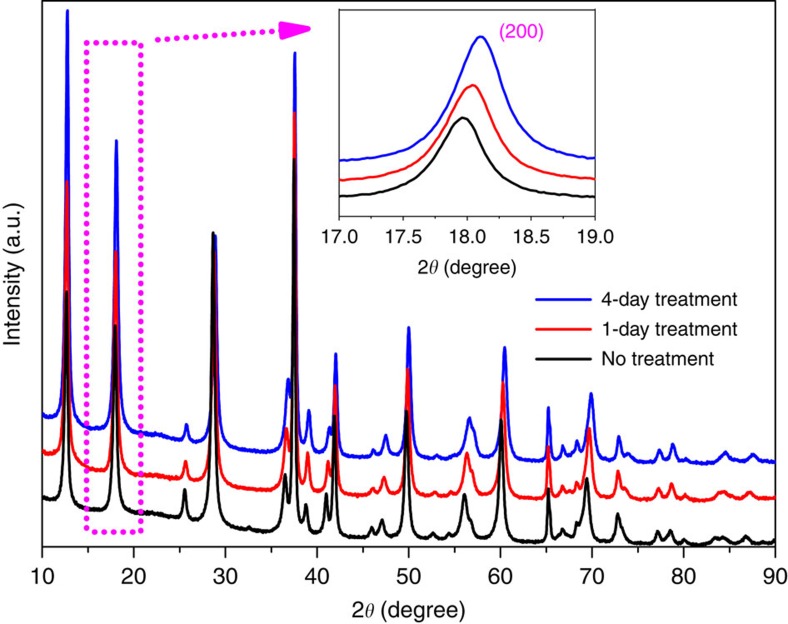
XRD patterns of the three groups of nanowires after acid treatment over different timescales. The inset image is the enlarged view of the α-MnO_2_ (200) peak, where the gradual peak shift toward higher angle direction with increase of treatment time is observed.

The structural and compositional analyses of the nanowires after HNO_3_ treatment are carried out using X-ray powder diffraction (XRD) with Rietveld refinement and analytical STEM with EDS. The XRD results are shown in Fig. 2 with the refined lattice parameters and compositions shown in Supplementary Table 1. It can be seen that the α-MnO_2_ phase is well maintained during the acid treatment as no extra peak generation or elimination is observed. However, with the increase of treatment time, all the peaks gradually shift toward higher angles, and this trend is more clearly demonstrated by the inset image showing the (200)_α-MnO_2__ peak. The Rietveld refinement in the table quantifies the trend of tunnel contraction during acid treatment by deriving lattice parameters *a*, *b* and *c*, where the anisotropic tunnel expansion (mainly along *a*–*b* plane) is indicated. The estimated K^+^ concentration further confirms that the tunnel contraction is essentially determined by the gradual removal of K^+^ from the tunnels.

**Figure 3:**
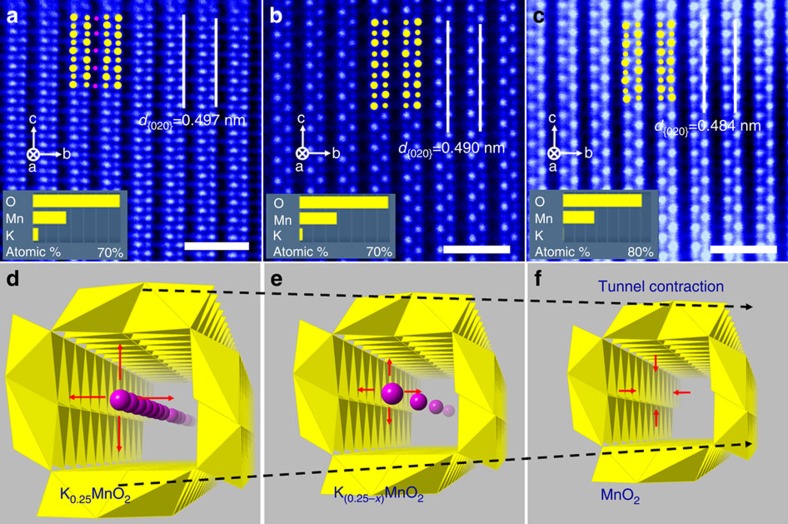
Demonstration of tunnel contraction during K^+^ removal by acid treatment. (**a**–**c**) False-coloured [100] HAADF images of α-MnO_2_ nanowires after (**a**) untreated, being treated with HNO_3_ for (**b**) 1 day, and (**c**) 4 days. Yellow spheres indicate Mn and pink spheres represent K^+^ inside 2 × 2 tunnels. The inset in each image shows the elemental concentrations of Mn, K and O based on EDS analysis of many nanowires. Scale bars in **a**–**c**, 1 nm. (**d**–**f**) The schematics of 2 × 2 tunnel cavity with different K^+^ concentrations showing the tunnel contraction when K^+^ ions are removed from the tunnel centre. The pink spheres indicate K^+^ on the centred 2a sites.

The atomic imaging of the tunnels with different K^+^ concentrations in Fig. 3 reveals that HNO_3_ treatment was effective in removing K^+^ from the 2 × 2 tunnels, and the K^+^ concentration can be controlled by altering the treatment time. After 1-day treatment, K^+^ has been partially removed from the nanowire, and the 2 × 2 tunnels are thus only partially filled. After 4-day treatment, α-MnO_2_ is essentially free of any K^+^ ions, as confirmed by both the HAADF image showing no K^+^ contrast (Fig. 3c) and the lack of any K^+^ signal in the EDS spectra shown in the inset. The compositions of α-MnO_2_ nanowires without treatment and after 1- and 4-day acid treatment were thus deduced to be K_0.25_MnO_2_, K_0.25−*x*_MnO_2_ and undoped MnO_2_, respectively. It is also notable that the 2 × 2 tunnels slightly contract after K^+^ removal, as indicated by the {020} spacing changing from 4.97 Å for the untreated nanowire to 4.84 Å for the 4-day treated nanowire. This tunnel contraction trend is clearly illustrated in Fig. 3d–f. Although the tunnel experiences significant volume change, the overall tunnelled structure of α-MnO_2_ is still maintained after K^+^ removal, enabling all these three types of nanowires to function as an intercalation cathode in a lithium ion battery.

There is a slight variation between XRD and atomic STEM imaging in determining the K^+^ concentration and lattice constants. This is understandable considering that STEM focuses on local atomic structure of a single nanowire, while XRD provides average bulk information including the surface lattice and structural defects. In our case, since the composition of the nanowires without HNO_3_ treatment is determined by counting the Mn and K atoms in the STEM image, which is mathematically more accurate than XRD, we have identified these nanowires as K_0.25_MnO_2_. This indicates that all 2a sites inside the 2 × 2 tunnels are occupied by K^+^ (ref. 26). The composition for 4-day treated nanowires is determined to be nominally MnO_2_ because both atomic STEM/EDS and Rietveld refinement hardly show any signal for K^+^. For 1-day treated nanowires, we express the composition using K_(0.25−*x*)_MnO_2_.

Previous studies suggest that the removal of K^+^ from 2 × 2 tunnels in acid solutions is explained by either the K^+^–H^+^ exchange mechanism^27^,^28^, Mn oxidation mechanism^23^,^29^ or the existence of both^30^. The critical difference between them is whether the Mn valence is increased or not. In our study, the dominant mechanism is found to be Mn oxidation to Mn^4+^ rather than K^+^–H^+^ exchange. The detailed analyses of Mn valence change using electron energy loss spectroscopy (EELS), X-ray photoelectron spectroscopy (XPS) are provided in Supplementary Figs 2 and 3, respectively, and the discussions are given in Supplementary Note 1. Both EELS and XPS confirm the existence of Mn^3+^ in the nanowires before acid treatment (K_0.25_MnO_2_), while a portion of Mn^3+^ gradually decreases with increase of treatment time. The average Mn valence gradually increases to Mn^4+^ as a result of K^+^ removal. The structural techniques of Raman, Fourier transform infrared (FTIR), nuclear magnetic resonance (NMR) and inductively coupled plasma optical emission spectroscopy (ICP-OES) (Supplementary Fig. 4 and related discussions in Supplementary Note 2) are also provided to explore the chemical composition evolution during acid treatment, where the possibility of H^+^ presence in the tunnels is found to be negligible, which again supports the postulate that K^+^–H^+^ exchange is not the dominant mechanism.

Before the electrochemistry analysis, the surfaces and thermal stability of the nanowires were characterized to check whether the nanowires are affected by the acid treatment. The Brunauer–Emmett–Teller (BET) analysis given in Supplementary Table 2 (detailed discussions in Supplementary Note 5) and SEM images in Supplementary Fig. 5 show no significant change in the surfaces of nanowires. Thermogravimetric analysis (TGA) and *in situ* TEM heating techniques are used to test the thermal stability of the nanowires after K^+^ removal from the tunnels, and the results and detailed analysis are shown in Supplementary Figs 6 and 7, respectively, while the discussions are given in Supplementary Note 3. The results confirm that the nanowires after K^+^ removal from the tunnels are thermally stable without any phase transition or tunnel collapse up to 350 °C.

### Effect of K^+^ on electronic and ionic conductivities

Our previous simulation work^17^ suggest that 2 × 2 tunnels are the preferred Li^+^ transport channels in pure α-MnO_2_. We have also reported previously that Li^+^ ions thermodynamically occupy the off-centred Wyckoff 8 h site inside each 2 × 2 tunnel rather than the tunnel centre^8^,^17^, which is also demonstrated by other groups^31^. Therefore, with K^+^ on the centred 2a sites and the tunnel expanded by K^+^, it is reasonable to expect the K^+^ concentration inside the 2 × 2 tunnels to affect both electronic and ionic transport and hence the electrochemical behaviour of an α-MnO_2_ cathode. To examine these effects, the electronic conductivity and Li^+^ diffusivity of α-MnO_2_ nanowires with different K^+^ concentrations were characterized using *in situ* TEM and EIS methods.

**Figure 4:**
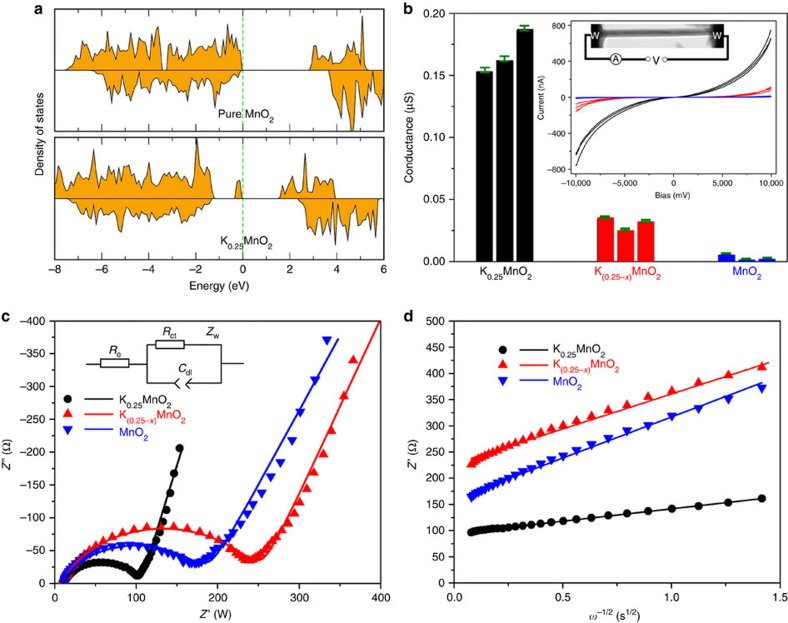
Conductivity measurements of α-MnO_2_ nanowires after acid treatment over different timescales. (**a**) Calculated electron density of states near the Fermi level region of K_0.25_MnO_2_ and pure MnO_2_. (**b**) Conductance of α-MnO_2_ nanowires with different K^+^ concentrations. Three nanowires were tested for each K^+^ concentration, as indicated by different coloured bars. The inset shows the *in situ* TEM experimental setup and the *I*–*V* responses of the nine α-MnO_2_ nanowires. (**c**) Impedance spectra (Nyquist plots) and (**d**) linear fitting to *Z*′ versus *ω*^−1/2^ plots in the low frequency range (<25 Hz) of the electrodes with K_0.25_MnO_2_, K_0.25−*x*_MnO_2_ or undoped MnO_2_ as the active material. The scatter points are the experimental data and the lines represent the simulation results using the equivalent circuit shown in **c**.

The electronic structure was also examined by DFT simulations and also by direct electrical probing within the microscope, the results of which are given in Fig. 4a,b. This computational work complements and extends previous DFT studies^8^,^16^ on interstitial cation incorporation in α-MnO_2_. The simulated density of states (shown in Fig. 4a) indicates that pure α-MnO_2_ has a bandgap of ∼2.8 eV suggesting semiconductor behaviour, and agrees well with the reported value of Young *et al*.^16^ For K_0.25_MnO_2_, newly formed occupied states appear inside the original MnO_2_ bandgap, indicating mixed Mn^4+^ and Mn^3+^ in K_0.25_MnO_2_, which is compatible with previous reports^32^. This indicates that the presence of K^+^ inside the 2 × 2 tunnels can enhance the electronic conductivity of α-MnO_2_ through electron hopping between Mn^4+^/Mn^3+^.

For each K^+^ concentration, the conductivity of three nanowires was measured to test reproducibility. The *I*–*V* responses of all the nanowires with different K^+^ concentrations measured *in situ* in the microscope (inset of Fig. 4b) are nonlinear but symmetric in the low-bias regime. This can be ascribed to the Schottky barriers formed between the semiconducting nanowire and the metal (W) electrodes^33^. In the large-bias regime (8–10 V), the *I*–*V* curves exhibit a near-linear relationship, and the conductance (*G*) of the nanowires can be calculated according to *G*=d*I*/d*V* (ref. 34). *G* was calculated to be 0.15–0.19 μS for K_0.25_MnO_2_, 0.025–0.034 μS for K_0.25−*x*_MnO_2_ and 0.0011–0.0050 μS for undoped MnO_2_; the results are plotted in Fig. 4b, and details of the data fitting are provided in Supplementary Table 3. Because of the similar diameters and lengths of the selected nanowires used for testing, the conductivities of nanowires with different K^+^ concentrations should exhibit similar trends as the conductance. It can be seen that K_0.25_MnO_2_ has a conductivity about 40 times higher than that of pure α-MnO_2_, indicating the important role of tunnel cations in enhancing the electronic conductivity of α-MnO_2_ nanowires. The origin of the improved electronic conductivity of α-MnO_2_ containing K^+^ can be attributed to the electron hopping between heterovalent Mn pairs (Mn^3+^/Mn^4+^) induced by K^+^ doping^35^,^36^. Increasing the cation content will produce more regions inside the nanowire with mixed Mn^3+^/Mn^4+^ valence states, resulting in higher electronic conductivity^37^.

Li^+^ diffusion in α-MnO_2_ nanowires with different K^+^ contents was characterized by EIS, and the results are shown in Fig. 4c,d. Figure 4c shows the impedance spectra (Nyquist plots) of electrodes with K_0.25_MnO_2_, K_0.25−*x*_MnO_2_ or undoped MnO_2_ as the active material. In the low frequency region the real part of the impedance (*Z*′) is linear against the −1/2 power of the angular frequency (*ω*^−1/2^), and the slope is called the Warburg coefficient (*σ*). Linear fits to the *Z*′ versus *ω*^−1/2^ plots for the untreated and treated α-MnO_2_ electrodes are shown in Fig. 4d, and the corresponding *σ* values are listed in Table 1. In addition, the equivalent circuit (inset of Fig. 4c) was used to fit the spectra and the results are listed in Table 1. The charge-transfer resistance (*R*_ct_) increased after K^+^ removal, indicating that doping with K^+^ enhances lithium intercalation near the electrode/electrolyte interface.

The chemical diffusion coefficient of Li ions 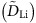 inside an electrode can be derived using the following equation^38^,


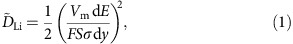


where *V*_m_ is the molar volume of the active material, *F* is the Faraday constant, *S* denotes the active surface area of the positive electrode and d*E/*d*y* is the slope of the open circuit potential versus the Li^+^ concentration *y* in Li_*y*_K_*x*_MnO_2_. The value of d*E/*d*y* was obtained from galvanostatic intermittent titration technique (GITT) measurements. The specific surface areas were obtained from BET results (Supplementary Table 2), with 28.2 m^2^ g^−1^ for K_0.25_MnO_2_, 26.8 m^2^ g^−1^ for K_0.25−*x*_MnO_2_ after 1-day acid treatment and 26.7 m^2^ g^−1^ for pure MnO_2_ after 4-day treatment, respectively. The measured values agree well with reported specific surface areas for hydrothermally synthesized α-MnO_2_ nanostructures which is in the range of 20–40 m^2 ^g^−1^ (refs 12, 39, 40, 41). The values of 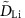 are listed in Table 1 showing that the chemical diffusion coefficient of Li^+^ is reduced by removal of K^+^. In addition, extending the time of acid treatment from 1 day to 4 days leads to a three-orders-of-magnitude decrease in the value of 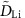. These results directly confirm that K^+^ in α-MnO_2_ tunnels facilitates diffusion of Li^+^. Although there are currently no measured diffusion data on Li/K–MnO_2_ for direct comparison, the magnitudes of 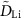 are comparable to other electrode materials; for example, experimental diffusion coefficients of 10^−8^–10^−12^ cm^2^ s^−1^ have been reported for Li^+^ diffusion in layered oxide cathodes such as LiCoO_2_ and Li(Ni,Mn,Co)O_2_ (refs 42, 43, 44). The increase of Li^+^ diffusivity by the addition of K^+^ can be attributed mainly to the expansion of the tunnel cavity by the large centred K^+^ cation at 2a sites. The improved *e*^−^ conductivity is also beneficial for Li^+^ conductivity in the tunnels with the presence of K^+^.

### Rate performance of α-K_*x*_MnO_2_ cathodes

Since the presence of tunnel cations improves both the electronic conductivity and Li^+^ diffusivity of α-MnO_2_, the rate performance of a Li/α-MnO_2_ battery would be expected to be enhanced when K^+^ occupy the tunnels. Before we examined the rate performance, the tunnel stability as well as the stability of K^+^ inside the tunnels during repetitive cycling are characterized. Supplementary Fig. 8a–c shows the morphology and phase analysis of three groups of nanowires (K_0.25_MnO_2_, K_0.25−*x*_MnO_2_, pure MnO_2_) after 100 battery cycles (Li/α-K_*x*_MnO_2_ coin cells). Supplementary Fig. 8d shows the EDS quantification of K^+^ concentrations inside the tunnels before and after battery cycles for the electrode initially composed of K_0.25_MnO_2_ nanowires. The K^+^ concentration in the tunnels is slightly reduced after cycling (probably lost into the electrolyte), while the majority of K^+^ remains inside the nanowires. All the nanowires, including those with K^+^ (partially and totally) removed, retain their tunnelled α-MnO_2_ structure with no obvious morphology change during battery cycling. Therefore the good stability of K^+^ ions in the tunnels can retain their effect on the battery performance upon continuous cycling.

To investigate the effect of K^+^ concentration on the first cycle performance, the galvanostatic discharge/charge curves for three groups of nanowires are shown in Supplementary Fig. 9. While all exhibit a discharge reaction around 2.5 V versus Li/Li^+^, the discharge capacity of the nanowires without acid treatment (143 mAh g^−1^) is relatively lower than that of the treated nanowires (157 and 170 mAh g^−1^), which can be ascribed to the addition of inactive mass of K^+^ into the electrode. Upon charge, however, the capacity of the treated nanowires is lower than the capacity of the nanowires with no treatment.

**Figure 5:**
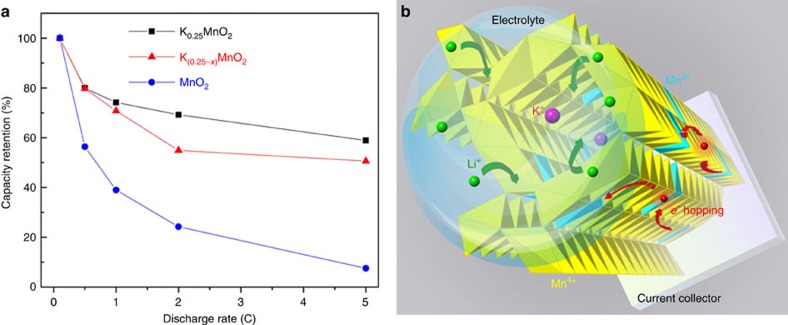
The effect of K^+^ concentration on rate performance of α-MnO_2_ nanowires as the lithium ion battery cathode. (**a**) Capacity measurements during the first discharge process of Li/K_0.25_MnO_2_, Li/K_0.25−*x*_MnO_2_ and Li/MnO_2_ based batteries at discharge rates of 0.1, 0.5, 1, 2 and 5 C. The 0.1 C capacities were normalized to 100% for a straightforward comparison between different C rates. (**b**) Three-dimensional schematic showing how K^+^ doping may facilitate Li^+^ insertion into the tunnelled electrode coated on current collector. The possible atomic arrangement inside the tunnels is calculated and explained in Supplementary Note 6.

To study the rate performance, the first discharge capacity of a Li/α-MnO_2_ battery under different current rates (0.1, 0.5, 1, 2 and 5 C) was measured for the three groups of nanowires. To make the comparison more straightforward, the discharge capacity at 0.1 C was normalized to 100%. From Fig. 5a, it can be seen that the first discharge capacities of cathodes made of K_0.25_MnO_2_, K_0.25−*x*_MnO_2_ and undoped MnO_2_ were all reduced when the C rate was increased from 0.1 to 5 C. The capacity retention behaviour under high discharge currents, however, varied with different K^+^ concentrations. Li/K_0.25_MnO_2_ exhibited 62% capacity retention at 5 C, while Li/K_0.25−*x*_MnO_2_ retained only 54% capacity and Li/MnO_2_ showed capacity retention as low as 8% at 5 C. The cycling-rate performance of the three groups of nanowires is shown in Supplementary Fig. 10 with the detailed discussions given in Supplementary Note 4. While the capacity retention is similar for the three groups at lower current, it shows gradually better capacity retention at higher currents (2 C and 5 C) for the nanowires with the higher K^+^ concentration. As discussed earlier, the presence of K^+^ in tunnelled α-MnO_2_ not only improves electronic conductivity by boosting e^−^ hopping *via* Mn^3+^/Mn^4+^ couples, but also enhances Li^+^ diffusivity. The detailed mechanism is illustrated in Fig. 5b.

Our investigation opens up further research opportunities in this area. For example, although not a trivial task, future atomistic simulations could be used to explore Li-ion diffusion in these nanowire structures with large cations inside. It would also be interesting to explore replacing K^+^ with other cations such as Ag^+^, Ba^2+^, to examine models related to cation-tunnel interactions, such as charge-switching states proposed by Young *et al*.^16^, and to study the effect of trace amounts of H^+^ *via* sensitive techniques such as quasi-elastic neutron scattering. Currently, we have found no experimental evidence showing the direct capacity contribution from Li^+^ intercalation into 1 × 1 tunnels, although future work could explore this possibility as a competing mechanism to insertion into 2 × 2 tunnels where large cations reside.

## Discussion

In this study, the effect of tunnel cations (K^+^) on the electrochemical performance of α-MnO_2_ cathodes was examined using a powerful combination of analytical ACSTEM, *in situ* TEM, electrochemical testing and *ab initio* modelling. α-MnO_2_ nanowires with different K^+^ concentrations were prepared and imaged at sub-ångstrom resolution to determine the structure of tunnels as well as the location and content of K^+^. It was found that the presence of K^+^ inside the 2 × 2 tunnels of α-MnO_2_ nanowires improves both their electronic conductivity and Li^+^ diffusivity. These enhancements facilitate favourable electrode kinetics, and thus result in good rate performance of Li/α-MnO_2_ based batteries.

The results of our systematic study provide a valuable framework for the rational selection of tunnel cations and their concentrations to improve the rate performance of tunnel-based intercalation electrodes. In addition, the favourable effect of K^+^ incorporation suggests that further exploration of tunnel-based cathodes with new battery chemistries based on Na^+^, Mg^2+^, and Al^3+^ ions is also warranted.

## Methods

### Materials synthesis and composition control

α-MnO_2_ nanowires were synthesized hydrothermally using KMnO_4_ and MnSO_4_ as reactants in aqueous solution^14^. Specifically, 0.9878, g of KMnO_4_ and 0.4226, g of MnSO_4_·H_2_O were dispersed in 80 ml of deionized (DI) water under constant stir for 30 min to form a purple solution. The obtained slurry was then transferred to a 100 ml Teflon-lined stainless steel autoclave, sealed and heated at 160 °C for 12 h. The synthesized nanowires were first separated from the solution by centrifugation, then washed with DI water and ethyl alcohol, and finally dried in air at 60 °C for 12 h. During the synthesis process, K^+^ doped the initially formed 2 × 2 tunnels and remained trapped inside the tunnelled structure.

The control of K^+^ concentration inside the 2 × 2 tunnels was achieved by acid treatment using concentrated HNO_3_ (ref. 45). The as-synthesized nanowires were first soaked in HNO_3_ solution accompanied by magnetic stirring (800 r.p.m. at 60 °C) for 1 and 4 days. This procedure was followed by repeated washing of the nanowires with DI water until the solution was neutralized (pH≈7). These nanowires were finally heated at 280 °C in air. According to literature^7^,^46^, 280 °C dry heating is efficient in removal of any residual water in the tunnels, which is also confirmed by the TGA results in Supplementary Fig. 6 for the thermal stability analysis.

### Materials characterization

Cross-sectional specimens were prepared using an ultramicrotome (Leica UCT). The nanowires were first mixed with cold-mounting epoxy resin (EPOFIX, Electron Microscopy Sciences) before subjecting them to 10 min supersonic vibrations. The hardener (EPOFIX, Triethylenetetramine, Electron Microscopy Sciences) was then added to speed up the solidification process in air at 60 °C for 24 h. After solidification, the sample was placed under an Edge CraftTM diamond knife mounted in an ultramicrotome (Leica UCT) to be cut into slices with a feeding step size of 500 nm.

(S)TEM characterization was performed using an aberration-corrected JEOL JEM-ARM 200CF STEM equipped with a 200 keV cold-field emission gun, a HAADF detector and an Oxford X-max 80 SDD X-ray detector. HAADF images were acquired using a 22 mrad probe convergence angle and a 90 mrad inner-detector angle. EELS data were captured in a postcolumn Gatan Enfina EELS spectrometer with a 45 mrad collection angle. The spectra were fitted using Gaussian function.

XPS was obtained from a Kratos AXIS-165 Surface Analysis System with the spectra collected with a monochromatic Al Ka source (1,486.7 eV). For survey spectra, the data were collected at a pass energy of 80 eV (fixed analyser transmission mode), a step size of 1 eV and a dwell time of 200 ms. High-resolution regional spectra were collected with a pass energy of 20 eV (fixed analyser transmission mode), a step size of 0.1 eV and a dwell time of 500 ms.

Gas adsorption analysis was performed using a micromeritics porosity analyser (ASAP 2020), by subjecting solids to varying partial pressures of N_2_ at 77 K in a liquid nitrogen bath. All MnO_2_ samples were initially degassed on the instrument degassing station (180 °C at 10 × 10^−3^ torr for 4 h). The measurement consists of 30 points (15 adsorption and 15 desorption points). For consistent and reliable results, data in the linear range of *P*/*P*_o_ of 0.06–0.3 (*R*^2^=0.9999) was used to calculate the BET surface area.

TGA was done with a SDT-Q600 TGA/DSC instrument with 100 ng sensitivity under high purity nitrogen atmosphere. In this experiment, 2.1 mg of sample was poured in a clean aluminium pan, then the sample was kept at 40 °C for 30 min to stabilize the system and then it was heated to 800 °C with the rate of 10 °C min^−1^ continuously.

Raman spectra were obtained from a Renishaw 2000 or inVia microscope spectrometer with a He–Ne laser at an exciting wavelength of 633 nm. Raman spectrum collection was set up in a 180° reflective mode. Roughly 10% of the maximum 13 mW laser intensity was applied.

FTIR spectra were obtained from a Nicolet iS5 spectrometer (Thermo Scientific) with a diamond ATR window. All the measurements were performed in an Ar filled glove-box to avoid any negative effect. The spectra were collected in the wavenumber from 4,000 to 550 cm^−1^ with a resolution of 4 cm^−1^, under an automatic-baseline-correct mode with 20 iterations and a polynomial order of 2.

^1^H NMR experiments were performed with a HX MAS probe in a Bruker Avance 300 wide bore magnet. The samples were spinning at 60 kHz at 283 K. A pulse delay of 1 s was used. ICP-OES analysis was done in a Thermo iCAP 7,600 ICP-OES equipped with a dual view (Radial and Axial) optical emission spectrometer.

*In situ* TEM heating was done inside a JEOL 3010 TEM. The nanowires were first dispersed onto a TEM grid and then mounted to an *in situ* heating holder. After loaded into TEM chamber, the nanowires were heated to 450 °C with the rate of 10 °C min^−1^. Data were collected at 25, 200, 280, 350, 400 and 450 °C after maintaining samples at each specific temperature for 5 min.

### *In situ* TEM testing of the *I*–*V* response of single nanowire

The *I*–*V* response of a single α-MnO_2_ nanowire was obtained using a Nanofactory scanning tunnelling microscope holder inside a JEOL 3010 TEM operated at 300 KeV. An *in situ* scanning tunnelling microscope holder was utilized to manipulate single α-MnO_2_ nanowires into the desired position. All the tested nanowires have similar morphologies (50–60 nm in diameter and 500–700 nm in length). Each nanowire was first vertically attached to the tungsten (W) tip on one side of the circuit and moved toward the W tip on the other side using a piezoelectric control. To minimize contact effects, the W tips were electrochemically etched immediately before use. After a nanowire was connected to both W tips to form a complete circuit, a high voltage with a current of about 1 μA was applied to achieve a tight contact between the nanowire and the two tips.

### *Ab initio* computer modelling

Calculations were performed based on DFT with PAW potentials, as implemented in the VASP code^47^. The HSE06 hybrid functional with 25% Hartree–Fock exchange was applied, which has been demonstrated by others (ref. 16) to describe well d electron localization and therefore can reproduce correct mixed charge states of transition metal oxides. For bulk calculations a planewave basis set cutoff energy of 500 eV and a minimum grid of 2 × 2 × 7 k-points were used in the Brillouin zone. The structure including lattice parameters and atomic positions were relaxed until the residue force on each atom is <0.03 eV Å^−1^. The calculated lattice parameters for K_0.25_MnO_2_ agree well with experiment, as shown in Supplementary Table 4.

### Electrochemical testing

The electrode slurry was made of 80 wt% α-MnO_2_ nanowires, 10 wt% super P carbon and 10 wt% polyvinylidene difluoride binder in *N*-methyl-pyrrolidinone (NMP). The mixture was then cast onto an Al foil to make the electrode. The electrode was dried at 75 °C for 4 h, followed by thorough drying at 75 °C overnight under vacuum. Electrochemical measurement of the first discharge capacity was carried out using CR2032 coin cells with Li metal as the counter electrode, 1.2 M LiPF_6_ in EC/EMC (3:7 by weight) as the electrolyte, and Celgard 2325 membrane as the separator. Cells were cycled between 1.5 and 4 V at different rates.

Electrochemical impedance data were collected with a Solartron 1470E and 1451A cell testing system, using a 5 mV AC perturbation with frequencies ranging from 100 KHz to 50 mHz. A three-electrode system was used so that the spectra obtained correspond to the impedance of the cathode side only. A Li metal wire was used as the reference electrode and a piece of Li foil was used as the counter electrode. Spectra were fit according to the equivalent circuit in Fig. 4a, where *R*_e_ is the electrolyte resistance, *R*_ct_ is the charge-transfer resistance, *C*_dl_ is the double layer capacitance that takes the roughness of the particle surface into account and *Z*_W_ is the Warburg diffusive impedance^23^. The semicircle in the high frequency region of the impedance spectra can be assigned to the *R*_ct_*C*_dl_ elements, while the slope of curve in the low frequency region is governed by the Warburg diffusion of Li^+^ into the bulk of cathode particles^48^. GITT measurements were carried out with a negative current pulse at C/20 for 30 s, followed by relaxations for 5 h. This sequence of discharge pulse followed by a relaxation time was repeated until the potential reached 1.5 V. GITT curve is provided in Supplementary Fig. 11.

### Data availability

The authors declare that the data supporting the findings of this study are available within the paper.

## Additional information

**How to cite this article:** Yuan, Y. *et al*. The influence of large cations on the electrochemical properties of tunnel-structured metal oxides. *Nat. Commun.* **7,** 13374 doi: 10.1038/ncomms13374 (2016).

**Publisher's note:** Springer Nature remains neutral with regard to jurisdictional claims in published maps and institutional affiliations.

## Supplementary Material

Supplementary Information Supplementary Figures 1-11, Supplementary Tables 1-4, Supplementary Notes 1-6 and Supplementary References.

## Figures and Tables

**Table 1:** Transport properties derived from the impedance spectra (at *T*=25 °C).

**Samples**	***R***_**e**_ **(Ω)**	***R***_**ct**_ **(Ω)**	***σ*** **(Ω s**^**−1/2**^**)**	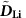 **(cm**^**2**^** s**^**−1**^**)**
K_0.25_MnO_2_	9.916	97.26	47.10	1.91 × 10^−12^
K_(0.25−*x*)_MnO_2_	8.198	246.3	133.5	7.16 × 10^−13^
Pure MnO_2_	7.261	182.2	160.5	2.82 × 10^−15^
