# Supplementary material for: The influence of large cations on the electrochemical properties of tunnel-structured metal oxides
Source: Nat Commun. 2016 Nov 21;7:13374. doi: 10.1038/ncomms13374 (PMC5473628; doi:10.1038/ncomms13374)
Supplement: Supplementary Information — Supplementary Figures 1-11, Supplementary Tables 1-4, Supplementary Notes 1-6 and Supplementary References. [file ncomms13374-s1.pdf]

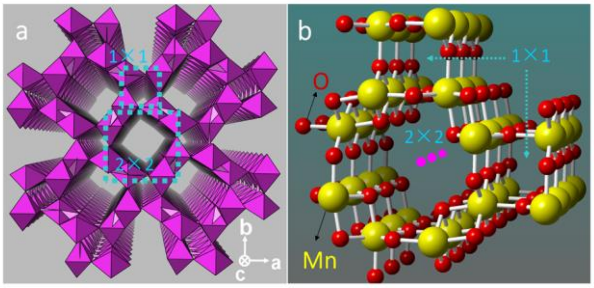

Supplementary Figure 1. Structural models for α-MnO_2. (**a**) Polyhedral model of α-MnO_2 along [001] zone axis with 1×1 and 2×2 tunnels indicated by the blue squares; (**b**) Atomic model showing one 2×2 tunnel and two adjacent 1×1 tunnels. The pink spheres refer to 2×2 tunnel stabilizers such as K^+, Ba^{2+} and NH_4^+.

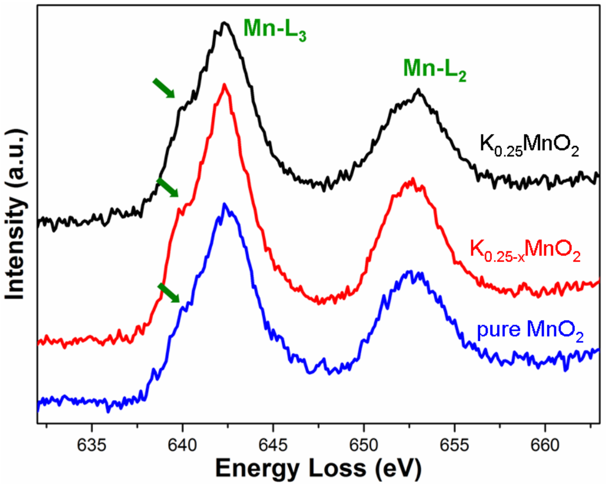

	$K_{0.25}MnO_2$	$K_{0.25-x}MnO_2$	pure MnO_2
L_2-L_3 (eV)	10.648 ± 0.024	10.596 ± 0.025	10.476 ± 0.033
Mn valence	**3.46**	**3.58**	**3.85**
L_3/L_2	2.092 ± 0.023	1.980 ± 0.035	1.837 ± 0.039
Mn valence	**3.72**	**3.84**	**3.97**

Supplementary Figure 2. The EELS results for three groups of nanowires ($K_{0.25}MnO_2$, $K_{0.25-x}MnO_2$, pure MnO_2). The green arrows indicate the existence of Mn^{3+}. The table on the btom shows quantification of Mn valence evolution during acid treatment using two methods, (L_2-L_3) and (L_2/L_3).

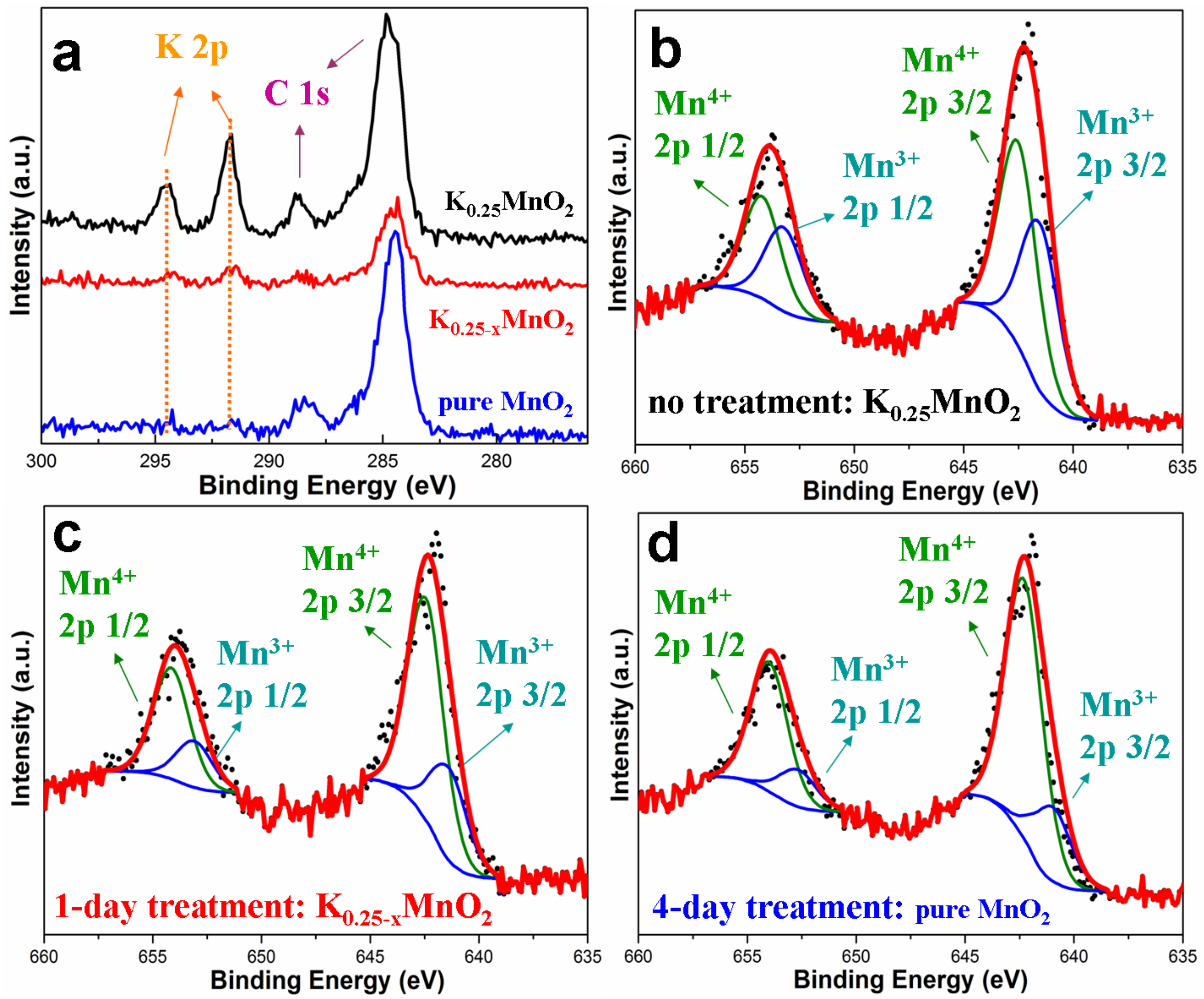

Supplementary Figure 3. XPS results of three groups of nanowires. (**a**) The XPS survey curves for three groups of nanowires (black for no treatment, red for 1-day treatment, blue for 4-day treatment). (**b-d**) The XPS results around Mn 2p peaks for three groups of nanowires.

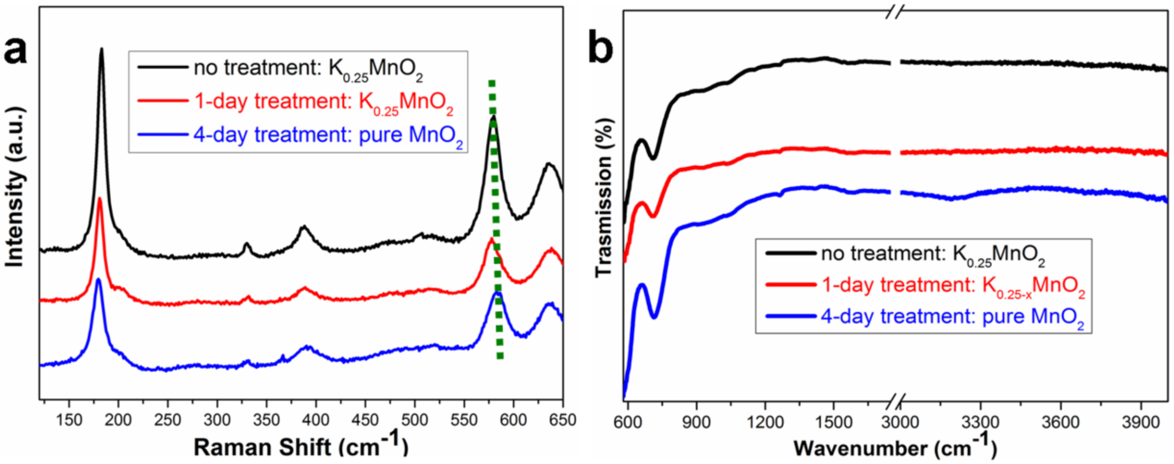

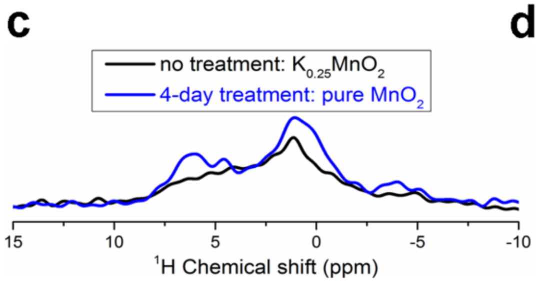

d

Sample	Mn content (ppm))	Mn content (ppm)	Mn content (ppm)	Averaged Mn content (ppm)
pure HNO_3 solution	**-0.0002**	**0.0003**	**-0.0037**	**0.003**
HNO_3 solution after treatment	**93.4140**	**92.3613**	**92.5640**	**92.780**

Supplementary Figure 4. Characterization of possible existence of H. (**a**) Raman spectra of the three groups of nanowires using a He-Ne laser, 632.8 nm. (**b**) FTIR of the three groups of nanowires recorded at room temperature. (**c**) 1H NMR for two groups of nanowires with K^+ fully doped and K^+ fully removed. (**d**) ICP-OES results showing the change of Mn content in HNO_3 solution before and after the acid treatment process.

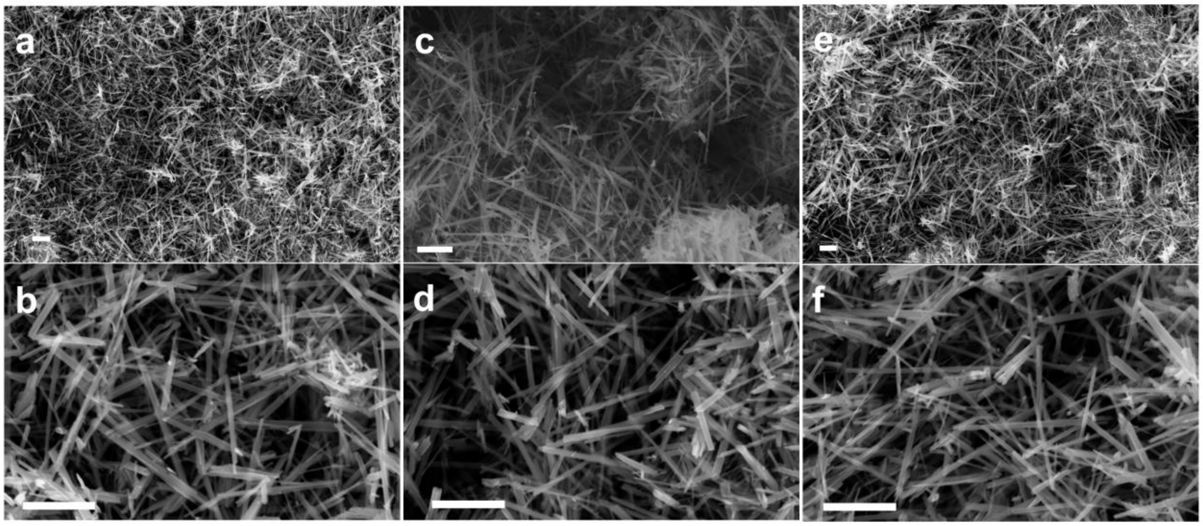

Supplementary Figure 5. Morphology evolution during acid treatment. SEM images of the three groups of nanowires with (**a**,**b**) for nanowries without acid treatment, (**c**,**d**) for nanowires after 1-day treatment, and (**e**,**f**) for nanowires after 4-day treatment. Scales bars for all images: 1 μm.

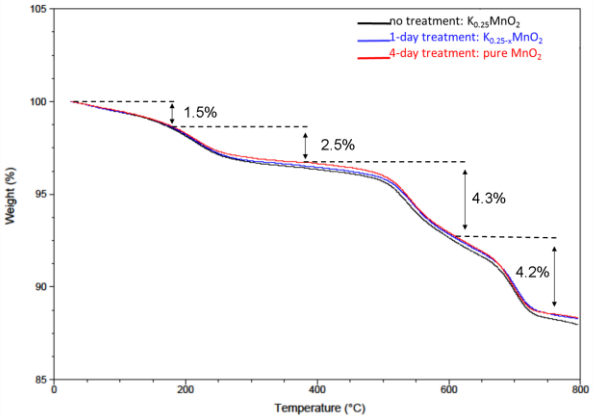

Supplementary Figure 6. TGA curves for the three groups of nanowires. Experiment was done in a N_2 atmosphere with a heating speed of 10 ^oC/min.

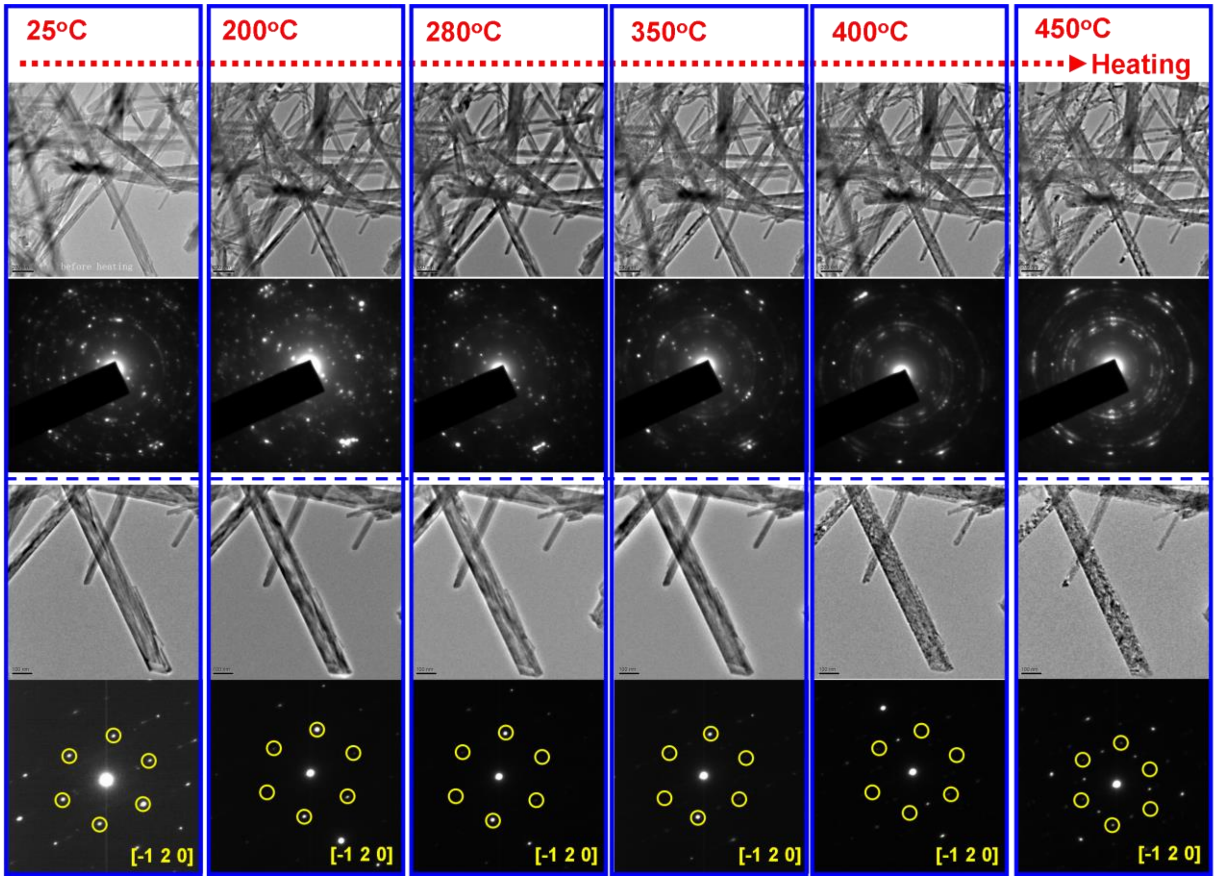

Supplementary Figure 7. Thermal stability study of α-MnO_2 nanowires. (Top two rows) *In situ* heating of several nanowires with their SAED recorded. (Bottom two rows) One nanowire is magnified for detailed phase and morphology analysis.

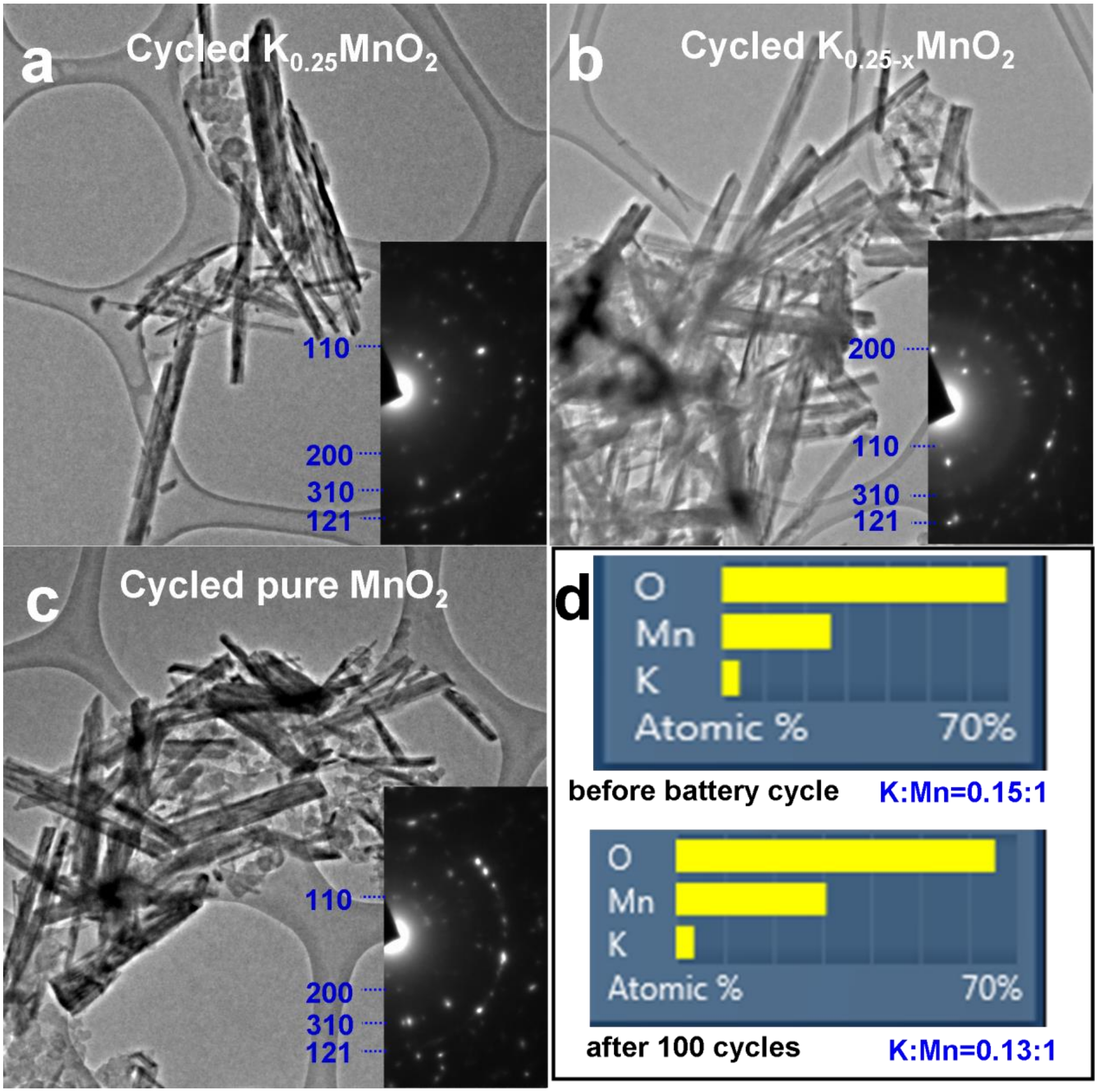

Supplementary Figure 8. ***Ex situ*** **TEM of cycled nanowires.** (**a-c**) TEM images and the corresponding SAED patterns for the nanowires after 100 cycles as lithium-ion battery cathodes. Indexing of the diffraction rings indicates that the nanowires consist of tetragonal α-MnO_2 and the tunnel structures are thus well maintained. (**d**) EDS analysis of the nanowires ($K_{0.25}MnO_2$ nanowires without acid treatment) before and after 100 battery cycles at 0.1 C. K^+ concentration in the nanowires is slightly affected by the battery cycling while the majority of K^+ are still within the nanowires. It shows that K^+ concentration in the tunnels is only slightly affected by the cycling, and a small amount of K^+ might be probably lost into the electrolyte while the majority of K^+ remains inside the nanowires.

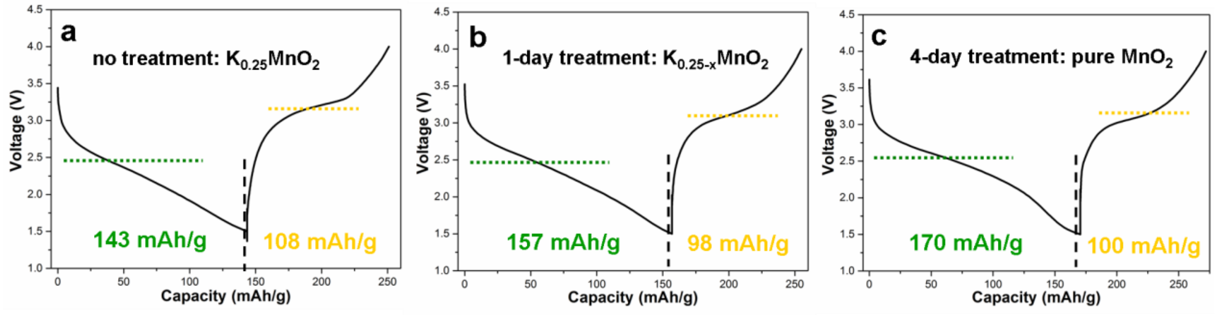

Supplementary Figure 9. The first discharge/charge profile for three groups of nanowires as lithium battery cathodes. The green line indicates the discharge reaction happening around 2.5 V for all three cells, while the yellow one indicates the charge reaction around 3.2 V for all three cells.

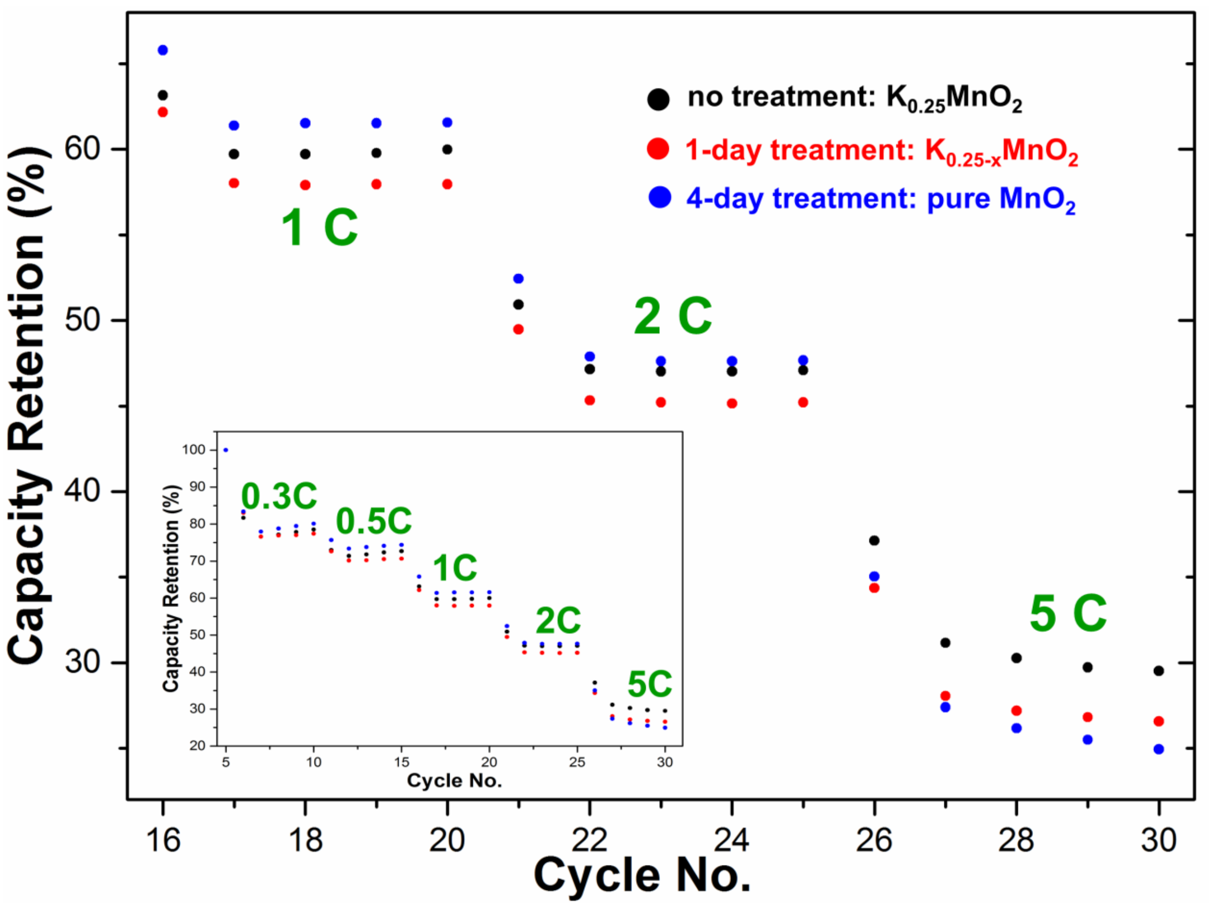

Supplementary Figure 10. Cycling-rate performance of the three groups of nanowires ($K_{0.25}MnO_2$, $K_{0.25-x}MnO_2$, pure MnO_2) as lithium battery cathodes at 1, 2 and 5 C. Each cycle is normalized to the discharge capacity at 0.1 C as shown in the inset.

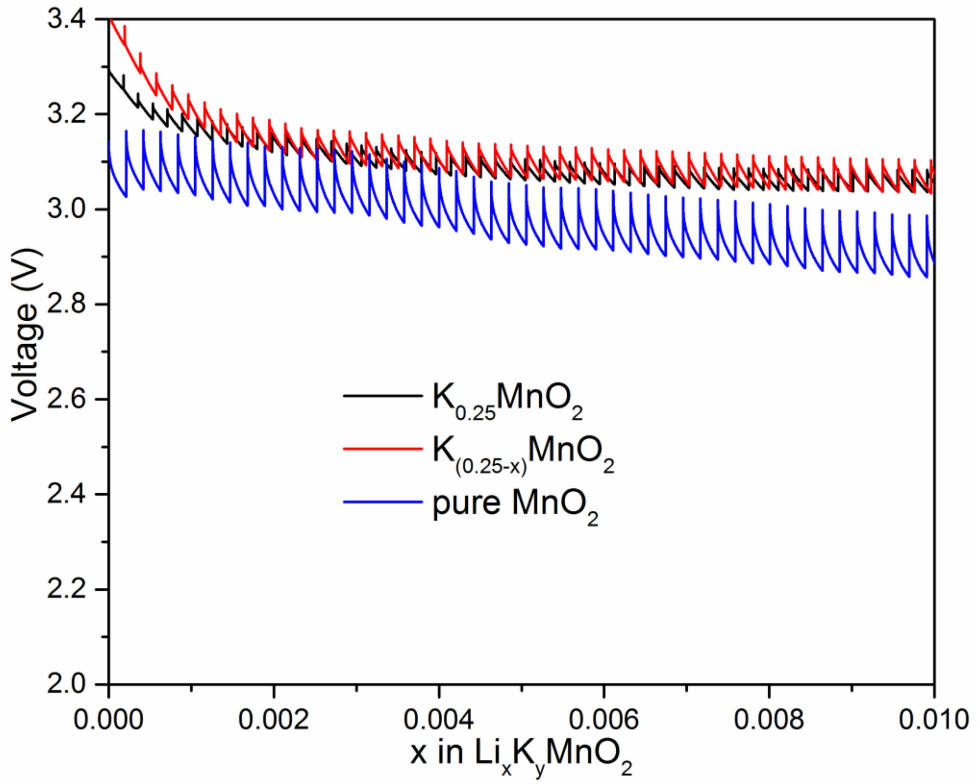

Supplementary Figure 11. GITT curves for the three groups of nanowires, where dE/dX is measured.

Supplementary Table 1. Calculated lattice parameters and compositions from the Rietveld refinement.

**Samples**	***a*=*b* (Å)**	***c* (Å)**	**Compositions**
**no treatment**	**9.82308**	**2.85443**	$K_{0.154}MnO_2$
**1-day treatment**	**9.78041**	**2.85355**	$K_{0.085}MnO_2$
**4-day treatment**	**9.74320**	**2.85346**	$K_{0.026}MnO_2$

Supplementary Table 2. BET surface area for the three groups of nanowires ($K_{0.25}MnO_2$, $K_{0.25-x}MnO_2$, pure MnO_2) (N_2 isotherm). Two experiments are carried out separately to minimize any operational errors.

	$K_{0.25}MnO_2$	$K_{0.25-x}MnO_2$	pure MnO_2
surface area (m^2 g^{-1}): 1st try	28.2	26.8	26.7
surface area (m^2 g^{-1}): 2nd try	30.8	29.7	31.0

Supplementary Table 3. Calculated conductance values for α-MnO_2 nanowires (at T = 25 ^oC) stabilized by different concentrations of K^+. The conductance values and error bars are for the linear fitting of all the curves in the region ranging from +8 V to +10 V.

Composition	Conductance (i.e. slope) (μS)	Error
**$K_{0.25}MnO_2$**	0.15253	0.00244
	0.16195	0.00303
	0.18614	0.00382
**$K_{(0.25-x)}MnO_2$**	0.03445	0.00112
	0.02485	0.00069
	0.03156	0.00041
**K_0MnO_2**	0.00496	0.00013
	0.00111	0.00003
	0.00122	0.00003

Supplementary Table 4. Calculated (DFT +U) and experimental lattice parameters of $K_{0.25}MnO_2$.

**Lattice Parameter**	**Expt Å**	**Calc Å**
**a**	**9.82**	**10.03**
**b**	**9.82**	**9.74**
**c**	**2.85**	**2.88**

Supplementary Notes

Supplementary Note 1: Discussion of Supplementary Figure 2 and Figure 3

According to the literature, there are two competing mechanisms explaining the removal of K^+ from the 2×2 tunnels of MnO_2 in an acid solution. One is K^+-H^+ exchange mechanism, and the other one is the redox mechanism. The detailed reaction paths for these two mechanisms were proposed by Feng et al.,[1] where the reactions are given as:

$$\{K_2\}[\square_{0.5}Mn^{IV}_{7.5}]O_{16}+2H^+ = \{H_2\}[\square_{0.5}Mn^{IV}_{7.5}]O_{16}+2K^+$$

for K^+-H^+ exchange, and

$$8\{K_2\}[Mn^{III}_2Mn^{IV}_6]O_{16}+32H^+ = 7\{\}[Mn^{IV}_8]O_{16}+16K^++8Mn^{2+}+16H_2O$$

for redox mechanism, where {}, [], and □ denote the (2×2) tunnel sites, octahedral sites for Mn, and octahedral vacant sites, respectively. The critical difference between these two mechanisms is whether Mn valence of the oxide is increased or not, which is however, not fully explored by the authors. Many groups also report the K^+ removal results are explained by either the K^+-H^+ exchange[2,3] or the redox mechanism[4,5]. Yet, there is no overall consensus as to which one is dominant due to the lack of a systematic study.

Our conclusion from the present study is that the K^+ removal from the 2×2 tunnels of MnO_2 is dominated by the redox mechanism, i.e. the Mn valence is gradually increased to Mn^{4+} with the removal of K^+. We systematically studied the Mn valence evolution for nanowires before HNO_3 treatment, after 1-day treatment, and after 4-day treatment using two different methods: electron energy loss spectroscopy (EELS) and X-ray photoelectron spectroscopy (XPS). If K^+ removal was controlled by the K^+-H^+ exchange mechanism, then the Mn valence would have been unchanged. The results of XPS and EELS both confirmed that there is an obvious trend of Mn oxidation (up to Mn^{4+}) with the gradual removal of K^+ from the tunnels, which is a solid proof that the K^+ removal is dominated by direct Mn oxidation instead of the K^+-H^+ exchange mechanism.

Supplementary Figure 2 shows the EELS data of the three groups of nanowires (top) with the quantification of Mn valence shown in the table (bottom). Three different signatures are used here to characterize the change of Mn valence. The green arrows indicate the existence of Mn^{3+} shoulder peak on the left side of the Mn^{4+} peak, which has been used as a signature of the Mn valence change.[6] The gradual decrease of the shoulder peak intensity during the acid treatment

confirms the gradual oxidation of Mn^{3+} to Mn^{4+} accompanying the removal of K^+ form the tunnels. Another signature is the energy difference between Mn white lines (L_2-L_3), which is known to be sensitive to the change of Mn valence.[6-8] The decrease of (L_2-L_3) value (shown in the bottom table of Supplementary Figure 2) during the treatment also confirms the oxidation of Mn. The third signature is the peak intensity ratio of L_3/L_2, which is also sensitive to Mn oxidation state.[9] The decrease of the L_3/L_2 ratio (shown in the bottom table of Supplementary Figure 2) during the treatment further confirms the oxidation of Mn. The quantification details are given in the table with reference to reported standard values[7].

Supplementary Figure 3 gives the XPS data of the three groups of nanowires with both the original curves and the fitted curves. Supplementary Figure 3a confirms the gradual removal of K^+ as evidenced by the decrease of K 2p signal during the treatment. Supplementary Figure 3b-d compare Mn 2p peaks for three groups of nanowires with emphasis on the quantification of Mn^{4+}/Mn^{3+} intensity ratio. After fitting the curves, the ratio of the integrated intensity of Mn^{4+} and Mn^{3+} peaks is (i) 1:1 for the nanowires without treatment, (ii) 2.5:1 for the nanowires after 1-day treatment, and (iii) 5: 1 for the nanowires after 4-day treatment. This indicates the Mn valence in K_xMnO_2 nanowires being increased from $Mn^{3.5+}$ (no treatment), to $Mn^{3.71+}$ (1-day treatment) and to $Mn^{3.83+}$ (4-day treatment). Note that XPS of the 4-day treated nanowires still shows a residual Mn^{3+} signal, which is probably due to the unsaturated Mn on the surface with 3+ valence states, which has been suggested by our recent work.[10]

In short, our XPS and EELS results on the Mn valence analysis are consistent with each other, leading to the conclusion that the K^+ removal process is dominated by the Mn oxidation mechanism rather than the K^+-H^+ exchange mechanism. So the possibility of H^+ presence in the tunnels is minimized.

Supplementary Note 2: Discussion of Supplementary Figure 4

We have considered the possibility of H^+ residing inside the 2×2 tunnels as a result of the K^+-H^+ exchange mechanism.[11] However, if we assume that one K^+ removal is compensated by one H^+ insertion into the tunnels, there should be no Mn oxidation. Our detailed Mn valence analysis clearly indicates Mn oxidation, and we therefore conclude that Mn oxidation is the dominant mechanism.

In addition, we have also carried out more we have done Raman spectra, Fourier transform infrared spectra (FTIR) and Nuclear Magnetic Resonance (NMR) spectra, and the results are shown as Supplementary Figure 4a, 4b and 4c, respectively. In addition, we also did Inductively Coupled Plasma Optical Emission Spectroscopy (ICP-OES) to see the change of Mn concentration in HNO_3 solution before and after the treatment, and the results are summarized in Supplementary Figure 4d.

In Supplementary Figure 4a, the band around 180 cm^{-1} is caused by external vibration deriving from the translational motion of MnO_6 octahedra, while the band around 390 cm^{-1} indicates the Mn-O bending vibrations.[12] The band around 570 cm^{-1} might refer to slight displacement of the oxygen atoms,[13] while the band around 630 cm^{-1} can be ascribed to Mn-O vibrations that are vertical to MnO_6 double chains.[14] These four strong bands as well as weak bands around 330 cm^{-1} and 510 cm^{-1} can be well indexed to be characteristic peaks for tunnel-based α-MnO_2 nanowires,[14] where Mn-O lattice vibrations within the MnO_6 octahedral frameworks are the origins. There is also an obvious blue shift for the band around 570 cm^{-1} as the sample is acid-treated, which according to literature, indicates the decrease of MnO_2 lattice dimension.[15] This finding also agrees with our XRD and STEM analyses (Figure 2 and Figure 3 in main paper) revealing tunnel contraction when K^+ are gradually removed form the tunnels during acid treatment.

In Supplementary Figure 4b showing the FTIR results, the peak around 720 cm^{-1} is attributed to the stretching vibration of Mn-O-Mn bonds in MnO_2. It is worth mentioning that while there are no obvious absorption bands around 3150 cm^{-1} for the samples without acid treatment and with 1-day treatment, a weak absorption peak can be vaguely captured for the sample after 4-day acid treatment. According to literature, this can be ascribed to the stretching vibration of OH^- bonded to Mn.[16] So this is probably an indication that H^+ go into the tunnels

and bonded to the host structure to some extent. However, compared to other reports showing similar OH- peaks,[14,16] the α-MnO2 nanowires after 4-day acid treatment in our work exhibit much lower absorption intensity around 3150 cm^{-1}. This is an indication that, although H^+ insertion could happen during the K^+ removal process, the amount of inserted H^+ is very limited.

Supplementary Figure 4c shows the NMR results for the nanowires without acid treatment and after 4-day acid treatment. One observation that merits attention is that the low signal to noise ratio of the 1H NMR spectra we obtained. The lack of 1H signal suggests that very little proton bear species (such as absorbed water and the OH functional group) exist in both samples. This is essentially why the baseline in both spectra wiggles.[17,18] This observation agrees with afore discussed Raman and IR spectroscopy that the amount of H^+ exchange is negligible.

A more straightforward evidence showing the existence of Mn oxidation mechanism dominating the K^+ removal process is given by ICP-OES in Supplementary Figure 4d. While the original HNO_3 solution shows no existence of dissolved Mn^{2+}, the solution used for 4-day acid treatment of $K_{0.25}MnO_2$ indeed shows significant increase in the Mn^{2+} concentration. This finding reasonably confirms the existence of the redox reaction:[4,5]

$$8\{K_2\}[Mn^{III}{}_2Mn^{IV}{}_6]O_{16} + 32H^+ = 7\{\}[Mn^{IV}{}_8]O_{16} + 16K^+ + 8Mn^{2+} + 16H_2O$$

where a small portion of lattice Mn are dissolved into the solution *via* Mn^{3+} disproportionation reaction.

So combining the analyses of Raman, FTIR, NMR and ICP-OES, we could reasonably conclude that the K^+ removal process is dominated by Mn oxidation process rather than the H^+-K^+ exchange mechanism.

Supplementary Note 3: Discussion of Supplementary Figure 6 and Figure 7

We have excluded the possibility of residual water inside the tunnels by heating the nanowires at 280 ^oC in dry air (right after acid treatment) to remove any absorbed water before the battery electrode lamination. According to previous studies, 280 ^oC is high enough to remove any tunnel water.[19,20] To confirm the removal of water as well as the thermal stability, we also carried out Thermogravimetric Analysis (TGA) on the three groups of nanowires, with the results shown in Supplementary Figure 6. The linear drop below 200 ^oC should be ascribed to the loss of surface water from the nanowires, which could start from 40 ^oC to 190 ^oC.[21] This value is small due to the fact that the nanowires had been preheated at 120 ^oC in a vacuum environment for 24 hours before the TGA experiment. An obvious weight drop of 2.5% starting from 200 ^oC to 300 ^oC should be caused by the loss of chemically bound water.[21,22] This also aggress with the reports demonstrating that 280 ^oC is high enough to remove all structural water in α-MnO_2.[19,20] Another weight drop of 4.3% from 500 ^oC to 600 ^oC should be caused by the loss of lattice O with the phase transition from α-MnO_2 to Mn_2O_3.[23] The drop of 4.2% after 700 ^oC can be ascribed to be the second loss of lattice O with the transition from Mn_2O_3 to Mn_3O_4.[23]

The in situ heating experiment was performed on a TEM heating stage, and the results are shown in Supplementary Figure 7. This real time study focused on tens of nanowires with one single nanowire targeted for the high-mag TEM imaging and diffraction. The selective area electron diffraction (SAED) pattern from these nanowires confirms that the tetragonal α-MnO_2 phase is stable when heated to 280 ^oC based on indexing the polycrystalline pattern to be α-MnO_2. The unchanged morphology of the nanowires (and the targeted single nanowire) also confirms the thermal stability at 280 ^oC. When it is heated above 350 ^oC (to 450 ^oC), the SAED pattern of these nanowires is still indexed to be tetragonal α-MnO_2 phase, but the polycrystalline pattern becomes ring-like. In addition, the dynamic observation of one single nanowire shows that the nanowire's single crystalline diffraction pattern also generates some new diffraction spots after 350 ^oC. Both signatures indicate the gradual polycrystallization of individual nanowire above 350 ^oC, although the tetragonal phase is maintained. The morphology evolution of the single nanowire further confirms the polycrystallization behavior, which starts with the surface pulverization around 350-400 ^oC and extends into the inner parts of the nanowire at 450

^{o}C. The thermal-induced polycrystallization of α-MnO_2 nanowires is probably driven by the formation of crystal interfaces and twin boundaries that are fast developing at high temperature.

Both TGA and in situ heating inside TEM confirm that the nanowires are thermally stable at 280 ^{o}C. Note that the thermal stability of the nanowires above 350 ^{o}C is characterized to be different in TGA and the in situ heating. This is understandable considering that the oxides are easier to release O under a TEM vacuum environment than in the N_2-filled TGA atmosphere.

Supplementary Note 4: Discussion of Supplementary Figure 9 and Figure 10

To investigate the effect of K^+ concentration during the first cycle, the galvanostatic discharge/charge curves for the three groups of nanowires are shown in Supplementary Figure 9. While all three groups exhibit a discharge voltage around 2.5 V (*vs* Li/Li^+), the variation in the first discharge capacity is observed among three groups of nanowires. The nanowires after 4-day acid treatment have a capacity of 170 mAh g^{-1}, compared to the capacity of 143 mAh g^{-1} for the nanowires without acid treatment, which can be ascribed to the addition of K^+ that increases the inactive mass of the material. Upon charge, however, we observed the opposite trend. The capacity of the 4-day treated nanowires is lower than the nanowires without treatment.

Supplementary Figure 10 gives the rate performance of the three groups of nanowires during cycling. The theoretical capacity for $K_{0.25}MnO_2$ is 208 mAh g^{-1} with 1 C equaling 208 mA g^{-1}, for $K_{0.25-x}MnO_2$ (assuming x=0.125) is 255 mAh g^{-1} with 1 C equaling 255 mA g^{-1}, and for MnO_2 is 308 mAh g^{-1} with 1 C equaling 308 mA g^{-1}. The discharge capacity of each cycle has been normalized to the capacity measured at 0.1 C. While the capacity retention is similar for the three groups at lower current, it indeed shows higher capacity retention at higher current (2 C and 5 C) for the nanowires with higher K^+ concentration.

Supplementary Note 5: Discussion of Supplementary Table 2

It can be seen form Supplementary Table 2 that these two BET tests agree with each other roughly well. We also note that there is no obvious surface area increase after acid treatment, and that the slight variation of the measured values should be caused by sampling. In fact, if we assume all Mn^{3+} go through the disproportionation reaction, the total amount of dissolvable Mn only take 12.5% of all the lattice Mn, meaning that most of Mn still remain in the lattice to support the host tunneled structure. This has been confirmed by our XRD and TEM analyses (Figure 2 and Figure 3 in the main text) showing no sign of structural degradation or morphological change. So even if there is certain morphology and surface change, it might be within a very small range that can not be reflected by BET analysis. In addition, from Supplementary Table 2 showing two tests of the same sample, the variation can be as large as 4.3 m^2/g (for the same MnO_2 group), which indicates the possibility of BET to neglect the slight change of surface area after acid treatment. Therefore, we consider there is no significant difference of the surface area before and after acid treatment.

Supplementary Note 6: Calculation of atomic arrangement inside the tunnels

We set r_{Li+}=0.59 Å, r_{K+}=1.37 Å, r_{O2-}=1.21 Å following Young's paper[11] and establish the atomic model with *a*=*b*=9.82308 Å and *c*=2.85443 Å based on our XRD Rietveld refinement in Supplementary Table 1. Then the D_{O-O} (the shortest distance of two O atoms surrounding one 8h site) is calculated to be 3.66 Å, D_{K-O}=(the shortest distance of one K and one O surrounding one 8h site) is 4.64 Å. So the O-O distance allows a maximum free spherical space with r_{max}= (3.66-2×1.21)/2=0.62 Å, while the K-O distance allows a maximum free spherical space of r_{max}=(4.64-1.37-1.21)/2=1.03 Å. So the theoretical maximum sphere that can be inserted around one 8h site is limited by D_{O-O} and the resulted r_{max} is 0.62 Å, which is larger than that of Li^+ (0.59 Å). This is an indication that Li^+ can be inserted at 8h sites when K^+ already occupy the 2a sites.

Supplementary References:

1. Feng, Q., Kanoh, H. & Ooi, K. Manganese oxide porous crystals. *J. Mater. Chem.* **9**, 319-333 (1999).

2. Tanaka, Y., Tsuji, M. & Tamaura, Y. ESCA and thermodynamic studies of alkali metal ion exchange reactions on an α-MnO_2 phase with the tunnel structure. *Phys. Chem. Chem. Phys.* **2**, 1473-1479 (2000).

3. Kijima, N., Ikeda, T., Oikawa, K., Izumi, F. & Yoshimura, Y. Crystal structure of an open-tunnel oxide α-MnO_2 analyzed by Rietveld refinements and MEM-based pattern fitting. *J. Solid State Chem.* **177**, 1258-1267 (2004).

4. Kijima, N. *et al*. Synthesis, crystal structure, and electrochemical properties of hollandite-type $K_xTi_{1-y}Mn_yO_2$. *Solid State Ionics* **262**, 14-17 (2014).

5. Feng, Q., Kanoh, H., Miyai, Y. & Ooi, K. Alkali metal ions insertion/extraction reactions with hollandite-type manganese oxide in the aqueous phase. *Chem. Mater.* **7**, 148-153 (1995).

6. Schmid, H.K. & Mader, W. Oxidation states of Mn and Fe in various compound oxide systems. *Micron* **37**, 426-432 (2006).

7. Zhang, S., Livi, K. J. T., Gaillot, A. C., Stone, A. T. & Veblen, D. R. Determination of manganese valence states in (Mn^{3+}, Mn^{4+}) minerals by electron energy-loss spectroscopy. *Am. Mineral.* **95**, 1741-1746 (2010).

8. Yuan, Y. *et al*. Asynchronous Crystal Cell Expansion during Lithiation of K^+-Stabilized α-MnO_2. *Nano Lett.* **15**, 2998-3007 (2015).

9. Wang, Z. L., Bentley, J. & Evans, N. D. Mapping the valence states of transition-metal elements using energy-filtered transmission electron microscopy. *J. Phys. Chem. B* **103**, 751-753 (1999).

10. Yuan, Y. *et al*. Atomistic Insights into the Oriented Attachment of Tunnel-Based Oxide Nanostructures. *ACS Nano* **10**, 539-548 (2016).

11. Young, M. J., Holder, A. M., George, S. M. & Musgrave, C. B. Charge Storage in Cation Incorporated α-MnO_2. *Chem. Mater.* **27**, 1172-1180 (2015).

12. Polverejan, M., Villegas, J. C. & Suib, S. L. Higher valency ion substitution into the manganese oxide framework. *J. Am. Chem. Soc.* **126**, 7774-7775 (2004).

13. Ma, S.B., Ahn, K. Y., Lee, E. S., Oh, K. H. & Kim, K. B. Synthesis and characterization of manganese dioxide spontaneously coated on carbon nanotubes. *Carbon* **45**, 375-382 (2007).

14. Liang, S., Teng, F., Bulgan, G., Zong, R. & Zhu, Y. Effect of phase structure of MnO_2 nanorod catalyst on the activity for CO oxidation. *J. Phys. Chem. C* **112**, 5307-5315 (2008).
15. Chen, D. *et al*. Probing the charge storage mechanism of a pseudocapacitive MnO_2 electrode using in operando Raman spectroscopy. *Chem. Mater.* **27**, 6608−6619 (2015).
16. Tsuji, M. & Tanaka, Y. Spectroscopic study of a cryptomelane-type manganic acid exchanged by divalent transition-metal cations. *J. Mater. Res.* **16**, 108-114 (2001).
17. Sa, N. *et al*. Is alpha-V_2O_5 a cathode material for Mg insertion batteries? *J. Power Sources* **323**, 44-50 (2016).
18. Sa, N. *et al*. Structural Evolution of Reversible Mg Insertion into a Bilayer Structure of $V_2O_5{\cdot}nH_2O$ Xerogel Material. *Chem. Mater*. **28**, 2962−2969 (2016).
19. Johnson, C.S. Development and utility of manganese oxides as cathodes in lithium batteries. *J. Power Sources* **165**, 559-565 (2007).
20. Espinal, L. *et al*. Time-dependent CO_2 sorption hysteresis in a one-dimensional microporous octahedral molecular sieve. *J. Am. Chem. Soc.* **134**, 7944-7951 (2012).
21. Wang, S. *et al*. Effects of polyaniline coating of cryptomelane-type KMn_8O_{16} on electrochemical performance for lithium-ion batteries. *J. Nanopart. Res*. **16**, 2232-2241 (2014).
22. Liu, L., Feng, Q., Yanagisawa, K., Bignall, G. & Hashida, T. Lithiation reactions of Zn-and Li-birnessites in non-aqueous solutions and their stabilities. *J. Mater. Sci*. **37**, 1315–1320 (2002).
23. Hashem, A. M. A., Mohamed, H. A., Bahloul, A., Eid, A. E., Julien, C. M. Thermal stabilization of tin-and cobalt-doped manganese dioxide. *Ionics* **14**, 7-14 (2008).
